# Prediction Algorithm of Parameters of Toe Clearance in the Swing Phase

**DOI:** 10.1155/2019/4502719

**Published:** 2019-08-14

**Authors:** Tamon Miyake, Masakatsu G. Fujie, Shigeki Sugano

**Affiliations:** ^1^Graduate School of Creative Science and Engineering, Waseda University, Tokyo, Japan; ^2^Faculty of Science and Engineering, Waseda University, Tokyo, Japan

## Abstract

The adaptive control of gait training robots is aimed at improving the gait performance by assisting motion. In conventional robotics, it has not been possible to adjust the robotic parameters by predicting the toe motion, which is considered a tripping risk indicator. The prediction of toe clearance during walking can decrease the risk of tripping. In this paper, we propose a novel method of predicting toe clearance that uses a radial basis function network. The input data were the angles, angular velocities, and angular accelerations of the hip, knee, and ankle joints in the sagittal plane at the beginning of the swing phase. In the experiments, seven subjects walked on a treadmill for 360 s. The radial basis function network was trained with gait data ranging from 20 to 200 data points and tested with 100 data points. The root mean square error between the true and predicted values was 3.28 mm for the maximum toe clearance in the earlier swing phase and 2.30 mm for the minimum toe clearance in the later swing phase. Moreover, using gait data of other five subjects, the root mean square error between the true and predicted values was 4.04 mm for the maximum toe clearance and 2.88 mm for the minimum toe clearance when the walking velocity changed. This provided higher prediction accuracy compared with existing methods. The proposed algorithm used the information of joint movements at the start of the swing phase and could predict both the future maximum and minimum toe clearances within the same swing phase.

## 1. Introduction

Robotic technology for physical human-robot interaction has the potential to improve human locomotion. Moreover, robotic assistance can guide gait motion and provide direct somatosensory information. Robotic guidance is effective because the effects of training last longer when people instinctively modify their motion, compared with when they consciously modify their motion [1]. Assistance should be provided only when it is required because the human movement ability decreases when it is not actively used [2]. Hence, there is a need for developing adaptive robotic assistance technology that encourages maximum active patient participation.

The human-centered control of robotics for gait training is being investigated in an attempt to make robotic systems more human-friendly [3]. Gait training robots, such as ALEX and Lokomat, have an interaction force field controller, which allows patients to walk in a matter that is different from the desired trajectory determined for a healthy person [4–6]. LOPES II, which is an end-effector type robot, is able to switch between low and high mechanical impedance modes using admittance control [7]. These robots adapt to individual differences and adjust their reference trajectory to recover motor functioning for gait trajectory generation. Conventional algorithms are adaptive after human action, and assistance methods for determining the robotic parameters by previously predicting the gait motion have not yet been established.

Falling is one of the most serious problems with locomotion. The risk of falling encourages people to stay indoors, which leads to the weakening of their bodies. Moreover, tripping accounts for 53% of falling incidents [8]. Older individuals are in more risk of tripping while taking small steps because it is difficult for them to assess the height difference at the edges of rugs or carpets [9]. Toe clearance must be ensured to avoid falling and controlled to reduce the dispersion. The possibility of tripping occurs if the toe approaches the ground at an arbitrary point in the gait cycle. The prediction of toe clearance can reduce the risk of tripping. For robotic assistance to increase the toe clearance when it decreases in the gait cycle, a method of predicting toe clearance is required.

Calculation techniques with wearable sensors deriving the toe clearance have been developed mainly for ambulatory estimation and monitoring of the toe clearance without a camera system [10–14]. The integration of the inertial parameters of the inertial measurement unit (IMU), which consists of triaxial accelerometers and gyroscopes, was carried out to estimate the toe parameters [10–12]. The dedrifted integration of two wirelesses IMUs attached to the feet can estimate the foot clearance with an error of approximately 20 mm [12]. Owing to this large error, the integration method has a large limitation with regard to calculating the position. A machine learning method has been developed to estimate the gait parameters after the learning phase in each person [13–15]. Using machine learning with Gaussian functions and a hill-climbing feature-selection method, the root mean square error (RMSE) of 6.6 mm was estimated for young individuals [14]. In previous research, the parameters of toe clearance were predicted by a regression model [15]. To the best of our knowledge, Gaussian functions that were applied using acceleration features through the double differentiation of the toe position captured with a motion capture system could predict the minimum toe clearance most accurately (an RMSE of 3.7 mm) for one gait cycle ahead.

The existing prediction method has a limitation with regard to establishing robotic assistance that increases the toe clearance when it decreases, because the system does not use wearable sensors that can communicate with a robot controller. The estimation accuracy is lower when the wearable inertial sensor is used, compared with when the motion capture system is used to extract the input data. Moreover, the existing method is not sufficiently accurate for handling the toe clearance variability between the gait cycles. Additionally, it has been reported that the interquartile range of the minimum toe clearance is approximately 4.3 mm for young individuals and approximately 5.3 mm for older individuals [16]. Detecting a lower value for the minimum toe clearance with a probability of more than 50% may be difficult using this method. Hence, a more accurate toe clearance prediction method that uses wearable sensors to obtain the input data is required for robotic assistance.

We developed a prediction algorithm of minimum toe clearance using the angular information of the lower limb joints [17]. Our hypothesis is that the articular motion information at the lower limb joints at the time when people start to swing their leg is related to the future toe clearance because the toe motion is generated by the swing motion of the lower limb. People control their leg motion based on interjoint coordination, and the angular coordination maintains low dispersion at the limb end points [18]. Therefore, we assumed that the difference between the angular information in a certain phase is related to the difference of toe clearance among the gait cycles. Moreover, we assumed that adding the angular velocity and acceleration of the lower limb joint would be beneficial because these parameters contain information regarding the movement over time. Previous studies have investigated computational technology, such as accelerometers [19, 20], gyroscopes [21, 22], and IMUs [23], for the detection of foot-contact state using wearable sensors and machine learning strategies implementing support vector machines (SVM) [24], linear discriminant analysis (LDA) [25], Gaussian mixture model (GMM) [26], and hidden Markov model (HMM) [27, 28]. Notably, none of these methods can detect the characteristic points of phase change in the angular trajectory. In a previous work, we extracted the characteristic angular point with consideration to the change of synergy between the hip, knee, and ankle joints and only predicted the minimum toe clearance with higher accuracy [17]. However, the wearable sensor tends to deviate while people walk, and the sensed values always contain noises. Compensation is required for the deviation of the sensed values.

In this study, we established an algorithm to predict the characteristic toe clearance parameters in the swing phase using the angles, angular velocities, and angular accelerations of the lower limb joints. We applied machine learning-based regression with Gaussian functions to probabilistically predict the toe clearance with consideration to the noise of the input data. Additionally, we investigated the relationship between the number of training data and the prediction accuracy, and we evaluated the prediction algorithm to investigate whether our method could more accurately predict the toe clearance and detect the lower value of toe clearance.

## 2. Materials and Methods

**Figure 1:**
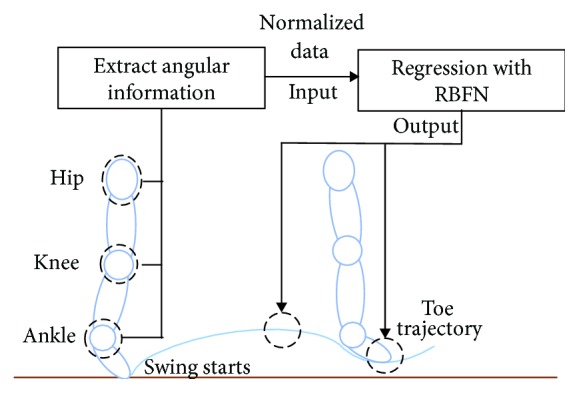
Overview of the dataflow of the proposed algorithm.

The proposed method consisted of extraction of input data and a regression algorithm using the radial basis function network (RBFN) to predict the characteristic parameters of the toe clearance as shown in Figure 1. The algorithm was designed to automatically extract the input data points in an earlier swing phase and normalize these input values to reduce the effect of the deviation of the sensor.

**Figure 2:**
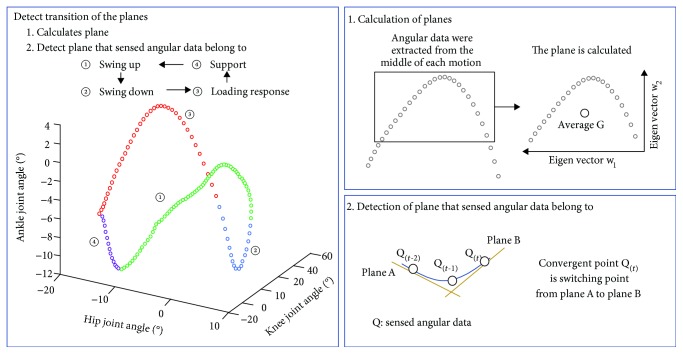
Overview of the algorithm for deriving the four planes in the angular space of the hip, knee, and ankle joints and for detecting the transitions of the planes.

The characteristic phase of input data was extracted with consideration to the synergy between the hip, knee, and ankle joints. The angular trajectory in the angular space is on the planes during walking [29], and the planes of these angles are different in the phases [30]. Detecting the change from the stance phase to the swing plane can be done more clearly in the planes, whereas detecting changes with the angle readings is difficult owing to the presence of noise and fluctuations in the angle range. As shown in Figure 2, the controller explores four planes in one gait cycle because the gait motion of the lower limb consists of the swing of the leg to lift the foot (swing up), the swing of the leg to prepare foot-ground contact (swing down), the loading response to absorb the shock of foot contact (loading response), and support for the body (support). First, the controller derives basis vectors of the planes by extracting parts of the angular data in each phase (block 1 of Figure 1). Second, the controller detects the switching points from the support phase to the swing up phase in an angular space so as to detect the time points when the swing phase starts (block 2 of Figure 1).

Parts of the angular data were extracted based on the hip angle to derive the planes for deriving the basis vectors of the planes (block 1 of the Figure 1). The maximum angle was defined as 100%, and the minimum angle was defined as 0%. First, the angular data were categorized as belonging to the motion of the swing up and were extracted when the hip motion was in more than 10% flexion and the knee joint was in flexion. Next, the angular data corresponding to the motion of the swing down were extracted when the knee joint extended and the hip flexion angle was within 30% after the swinging motion. Additionally, angular data corresponding to the loading response (i.e., dual-support phase) were extracted when the hip joint was in extension, the knee joint was in flexion, and the dorsiflexion angle of the ankle joint was less than 10% from the second minimum value. Finally, the parts of the angular data corresponding to the motion of supporting the body were extracted when the hip joint was in extension and the ankle joint was in dorsiflexion. The robot extracted the angular data in the middle of the swing or stance phase based on the hip angle readings.

Two basis vectors constituting the plane can be derived using principal component analysis (PCA) and the extracted parts of the angular data. The controller calculates the eigenvectors of the first and second components, which are the basis vectors of the plane, using PCA. The vector from the preprojection coordinates to the postprojection coordinates is orthogonal to the basis vectors of the plane. Moreover, the two eigenvectors **w**_1_ and **w**_2_ are perpendicular to each other. Using this relationship, the coordinates **P** on the plane are defined as follows:

(1) $$\begin{array}{c} P=a_{1}w_{1}+a_{2}w_{2}+G,\\ a_{1}=Q\cdot w_{1}-G\cdot w_{1},\\ a_{2}=Q\cdot w_{2}-G\cdot w_{2}, \end{array}$$

where *a*_1_ and *a*_2_ are the coefficients of the eigenvectors, **G** denotes the coordinates of the mean angle data, and **Q** denotes the sensed coordinates of the lower limb articular angular space before projection. *a*_1_ and *a*_2_ are calculated using the inner product of the eigenvectors and orthogonal vectors.

The algorithm calculates the distance from the preprojection coordinates to the postprojection coordinates on each plane to derive the switching points of the planes (block 2 of Figure 1). Additionally, the algorithm calculates the inner product between the unit vector from a previously sensed angular point to the currently sensed angular point and the unit vector from a previous projected point to the current projected point. The sensed angular data are recognized as a phase whose plane is closer to the data, compared with the other planes, when the distance is the local minimum and the inner product is more than 0.9.

**Figure 3:**
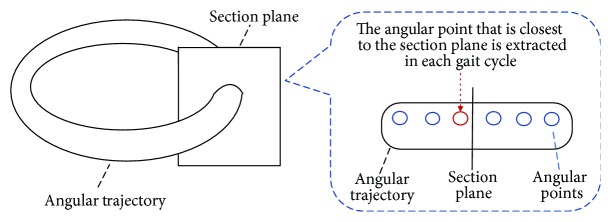
Extraction method of input values by finding the angular point that is the closest to the section plane when the gait state changes from the stance phase to the swing phase.

**Figure 4:**
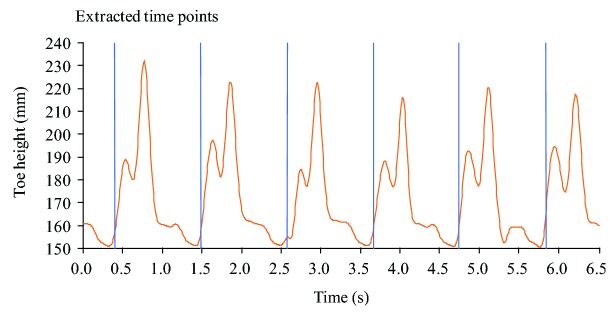
Gait phase detection result of extracting time points for use as input to the prediction algorithm.

The phase when the swing starts can be derived by observing whether the angular trajectory passes through the section plane that was previously calculated using the plane structure as shown in Figure 3. The section plane is calculated because it is difficult to detect the switching points of the planes in real time owing to the shifting of the plane during walking. First, the switching points from the plane of support to the plane of swing up were derived using the data obtained from the 20 gait cycles. Next, the section plane of the angular trajectory is calculated when the swing phase starts. The average switching point is estimated, and the normal vector of the section plane is calculated by deriving the vector from the detected switching points to the next sensed angular point. The orthogonal vector **v** of the normal vector can be calculated as follows:

(2) $$\begin{array}{c} v=\left[2bc,–ac,–ab\right], \end{array}$$

where *a*, *b*, and *c* denote the hip, knee, and ankle joint angles that constitute the normal vector, respectively. The basis vectors of the section plane are two orthogonal vectors of the normal vector, which is calculated by deriving the cross product between the first orthogonal vector and the normal vector. Finally, as shown in Figure 4, the angular points for the input data are extracted by finding the time point where the distance from the sensed angular point to the point projected onto the section plane is minimum.

**Figure 5:**
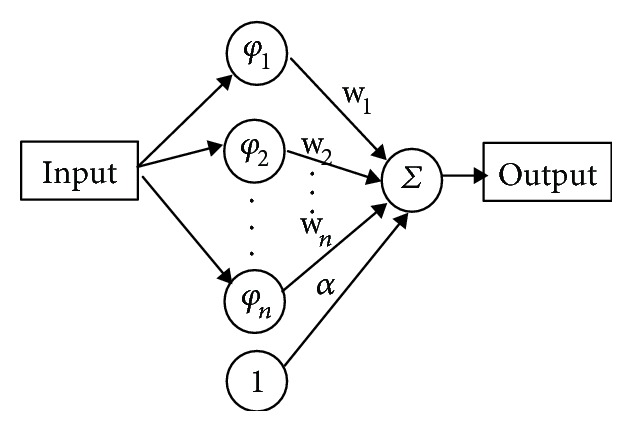
Structure of the radial basis function network (RBFN).

The parameters of toe clearance were calculated using the RBFN with Gaussian functions, as shown in Figure 5. The RBFN is the linear sum of the radial basis functions, such as the Gaussian functions, for nonlinear curve fitting. The RBFN consists of an input layer, a hidden layer with radial basis functions, and an output layer. This network calculates the distance between the vector of the input data and the centroids of each Gaussian, which are derived using the *K*-means clustering algorithm to partition the dataset into a predetermined number of groups according to the Euclidean distance. The RBFN structure is expressed as follows:

(3) $$\begin{array}{c} y=\overset{N}{\underset{k=1}{\sum }}w_{k}\exp\left(-\frac{{\left\|x-c_{k}\right\|}^{2}}{\sigma }\right)+\alpha , \end{array}$$

where **y** denotes the output vector, **w**_*k*_ is the weight vector, **x** is the input vector, **c**_*k*_ is the centroid vector, *N* is the number of RBF units, *α* is a variable coefficient, and *σ* is a variable related to the standard deviation of the Gaussian function. *σ* is derived as follows [31]:

(4) $$\begin{array}{c} \sigma =\frac{d_{\max}}{\sqrt[{m}]{Nm}}, \end{array}$$

where *d*_max_ denotes the maximum distance among the data and *m* is the dimension of the data.

The angles of the hip, knee, and ankle joints in the sagittal plane were sensed with wearable angle sensors. The angular velocity and angular acceleration of these joints were derived by differentiating the angles with a pseudo differential. The angles were smoothed using a low-pass filter (with a cutoff frequency of 6 Hz). The equation of the pseudo differential based on an s-plane to z-plane transformation is expressed as follows:

(5) $$\begin{array}{c} Y_{n}=\frac{X_{n}-X_{n-1}+T_{d}Y_{n-1}}{\Delta T}, \end{array}$$

where *T*_*d*_ denotes the time constant, Δ*T* denotes the sampling time, which was 8.33 ms, and *Y*_*n*_ and *X*_*n*_ denote the *n*^th^ differential value and *n*^th^ input value, respectively. In this study, *T*_*d*_ was considered 167 ms to differentiate the data whose frequency was lower than 6 Hz.

All input values were normalized to reduce the effect of attachment position deviation of the wearable angle sensors. The minimum values in the previous gait cycle were subtracted from the input values. Moreover, all input values were divided by their range of values in the first gait cycle in the training phase for RBFN so as to decrease the effect of the range of values.

## 3. Human Walking Experiment

Four healthy younger adults (three men and one woman; aged 27 ± 5 years, body weight 57 ± 13 kg, height 1.64 ± 0.13 cm) and two healthy older adults (two men; aged 65 ± 2 years, body weight 62 ± 1 kg, height 1.68 ± 0.03 cm) were recruited in the first experiment. Five healthy young adults (four men and one woman; aged 25 ± 3 years, body weight 58 ± 9 kg, height 1.63 ± 0.7 cm) were recruited in the second experiment. All of them did not have neurological injuries or gait disorders. Before the experiment, the subjects were provided with a detailed account of our experimental objectives and were informed that they could withdraw from the experiment whenever they desired, and we obtained their consent. This experiment was also approved by the institutional review board at Waseda University (No. 2017-085).

**Figure 6:**
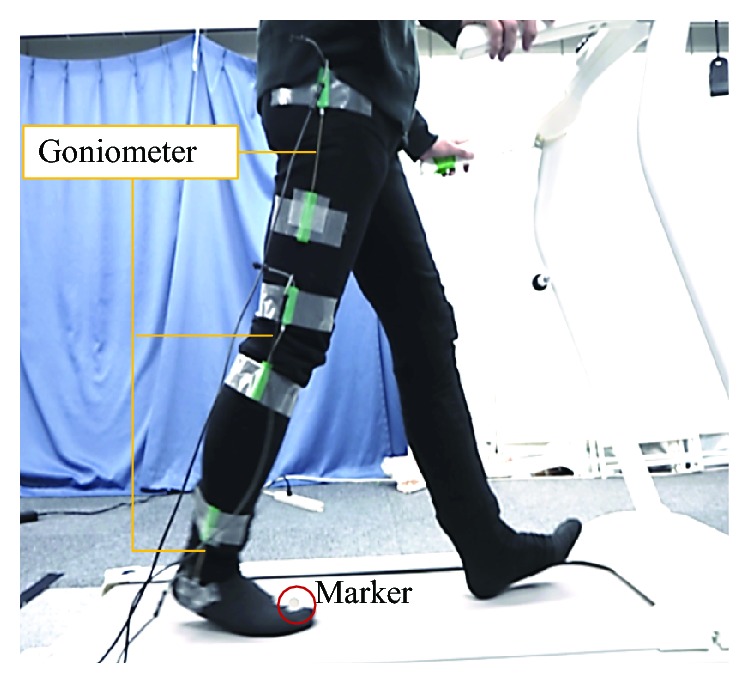
Experimental image of subjects walking on a treadmill.

Because the maximum values are an indicator of how high people raise their foot and the minimum values are an indicator of how high people can keep their foot above the ground, the maximum toe clearance in the earlier swing phase and the minimum toe clearance in the later swing phase were measured to give characteristic toe clearance data. The toe coordinates of the right foot were measured with a motion capture system (Raptor-E; Motion Analysis, Santa Rosa, CA, USA). The marker for the measurement was attached to the first metatarsophalangeal joint of the foot. The angles of the right hip, knee, and ankle joints were measured with goniometers (SG110 and SG150, Biometrics Ltd., Newport, UK), which are wearable angle sensors. The subjects walked on a treadmill as shown in Figure 6.

The 6 subjects were instructed to continue walking for 360 s at a preferred constant speed ranging from 2.1 km/h to 3.0 km/h in the first experiment. We investigated the number of training data points required for the RBFN to improve the prediction accuracy. We used 20 to 200 gait cycle data points for the training and 100 gait cycle data points for the RBFN test. The number of RBF units was set from two to twenty.

The 5 subjects walked for 600 s at 2.0 km/h, 2.5 km/h, and 3.0 km/h in the second experiment. The duration of walking at 2.5 km/h was 360 s, and the duration of walking at 2.0 km/h and 3.0 km/h was 120 s. We investigated whether the RBFN could predict the toe clearance if the walking speed changed. Approximately 160 cycle data of 2.5 km/h walking were used as the training data based on the result of the first experiment, and the 100 gait cycle data points of 2.0 km/h and 3.0 km/h were used as the test data. The number of RBF units was set from two to twenty. Moreover, we added the goniometers for a left leg in this experiment.

We derived the time from the time point where the system extracted the input data to the time points for the maximum and minimum toe clearances. We calculated the average time of all training data and the standard deviation to evaluate whether the system could have previously predicted both the maximum and minimum clearances.

**Figure 7:**
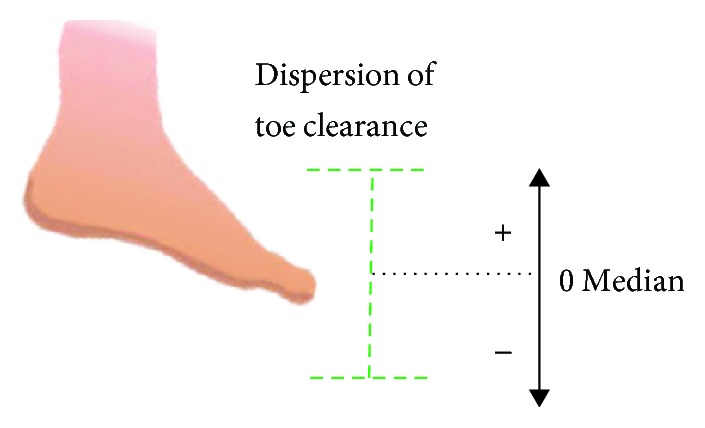
Normalization of toe clearance data.

We normalized the maximum and minimum toe clearance values by defining the average of the training data as zero as shown in Figure 7. The toe clearance values that were lower than the average were negative (minus sign), while the values that were higher than the average were positive (plus sign). We calculated the RMSE between the true value and the predicted value of the maximum and minimum toe clearances, as follows:

(6) $$\begin{array}{c} \text{RMSE}=\sqrt{\frac{\sum_{k=1}^{n}{\left(y_{k}-{\tilde{y}}_{k}\right)}^{2}}{n}}, \end{array}$$

where *y*_*k*_ denotes the true value, ${\tilde{y}}_{k}$ denotes the predicted value, and *n* is the number of data points.

Additionally, we estimated the accuracy percentage of the predicted data according to the accuracy of the plus or minus signs and counted the number of predicted values with the same sign as the true value, which was then divided by the total number of data points.

## 4. Results and Discussion

**Figure 8:**
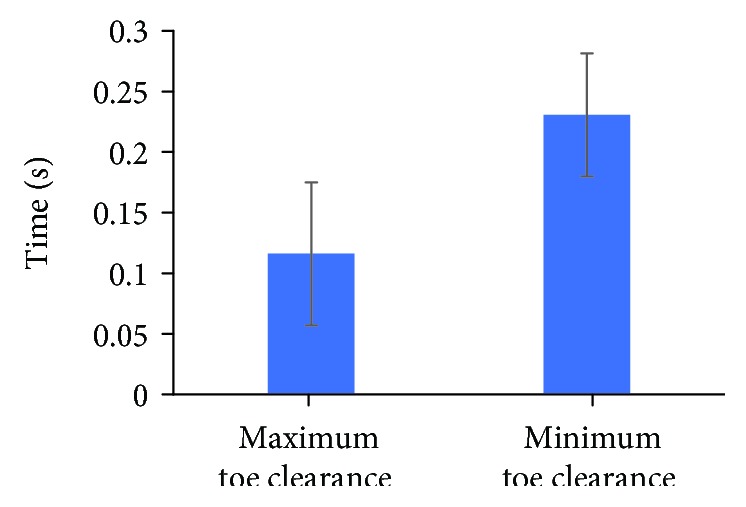
Time from points where the system extracted the input data to the points of the maximum and minimum toe clearances. The error bar indicates the standard deviation.

Figure 8 shows the time from the time points where the system extracted the input data to the time points of the maximum or minimum toe clearances. The input data were extracted 0.1 s before the toe clearance reached the maximum value in the earlier swing phase.

**Figure 9:**
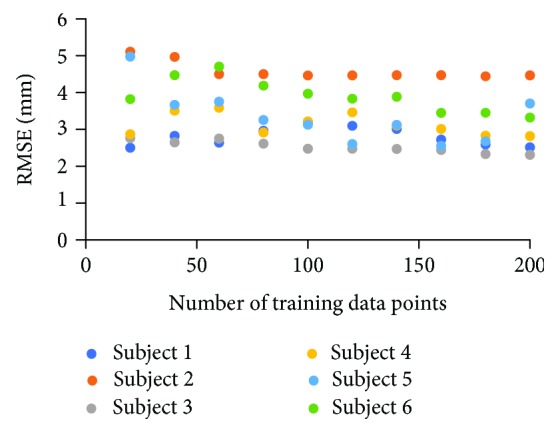
Prediction result for maximum toe clearance using 100 test gait data (RMSE).

**Figure 10:**
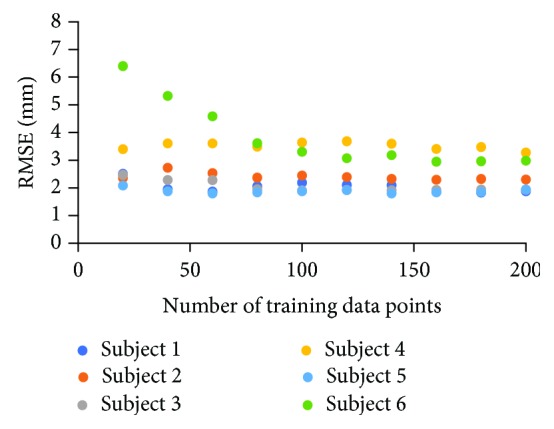
Prediction result for minimum toe clearance using 100 test gait data (RMSE).

Figures 9 and 10 show the RMSE between the true and predicted data for the maximum and minimum toe clearances corresponding to the number of training data points. The RMSE tended to decrease as the number of training data increased. Particularly, the RMSE was minimum when the number of training data points was 200 for subjects 1, 3, and 6. The other subjects had a minimum RMSE when the number of training data points was between 80 and 180. For the maximum toe clearance, the average minimum RMSE was 2.99 mm, and the lowest RMSE was 2.31 mm. For the minimum toe clearance, the average minimum RMSE was 2.34 mm, and the lowest RMSE was 1.79 mm. The number of RBF units that minimized the RMSE was approximately five.

**Figure 11:**
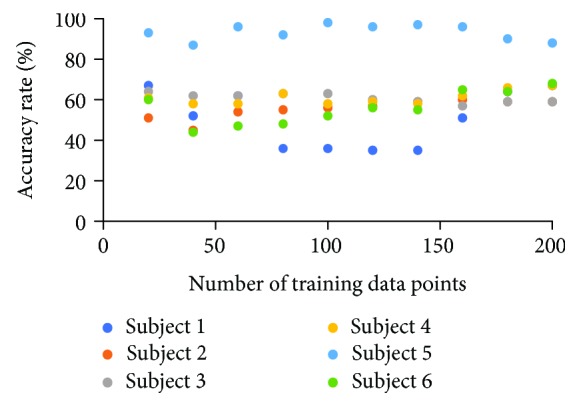
Prediction result for maximum toe clearance using 100 test gait data (accuracy rate).

**Figure 12:**
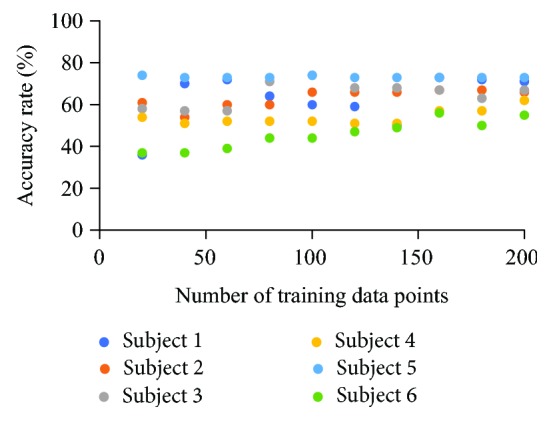
Prediction result for minimum toe clearance using 100 test gait data (accuracy rate).

Figures 11 and 12 show the accuracy rate of the predicted data for the maximum and minimum toe clearances corresponding to the number of training data points. The average accuracy rate was 71% for the maximum toe clearance and 68% for the minimum toe clearance.

Figure 8 shows the average time from the time points where the system extracted the input data to the time points where the maximum or minimum toe clearances were positive. This means that the proposed algorithm was able to extract the input data before the toe clearance reached its maximum value in the earlier swing phase. However, time was not always constant. The standard deviation was large compared with the time of the gait cycle, which was approximately 1.4 s in this experiment. The variance in the detection time plays a role in reducing the time. By improving the accuracy of phase detection, the prediction can be made earlier.

As shown in Figures 9 and 10, the RMSE between the real toe clearance measured by the motion capture system and the predicted toe clearance was the lowest between 80 and 200 training data points. Moreover, the accuracy rate tended to increase when the number of training data points increased. Therefore, a higher number of training data points tended to improve the prediction accuracy, presumably because it became easier to extract the characteristics of the input data space when more training data were provided. The RBFN clusters the input data and calculates the median values of each cluster in the training phase. The output values are determined according to the distance of the input data values from the median values of each cluster. If the number of training data points decreases, it becomes difficult to precisely determine the RBFN parameters because the effect of the input data noise increases. In this experiment, the clustering of training data points and the derivation of the median required approximately 100 to 200 training data points to reduce the variance and the effect of the noise that is always present in data.

As shown in Figures 9 and 10, the RMSE was 2.99 mm for the maximum toe clearance and 2.34 mm for the minimum toe clearance, which is a more accurate prediction compared with previous methods. The RMSE of the maximum toe clearance was higher than the RMSE of the minimum toe clearance, because the variance of the maximum toe clearance was higher than the variance of the minimum toe clearance. The individual difference between the RMSE tended to be higher as the variance of the toe clearance between cycles increased. The probability of detecting a value lower than that of the median toe clearance was higher than 68%; that is, the probability was higher than the probability of random detection.

**Figure 13:**
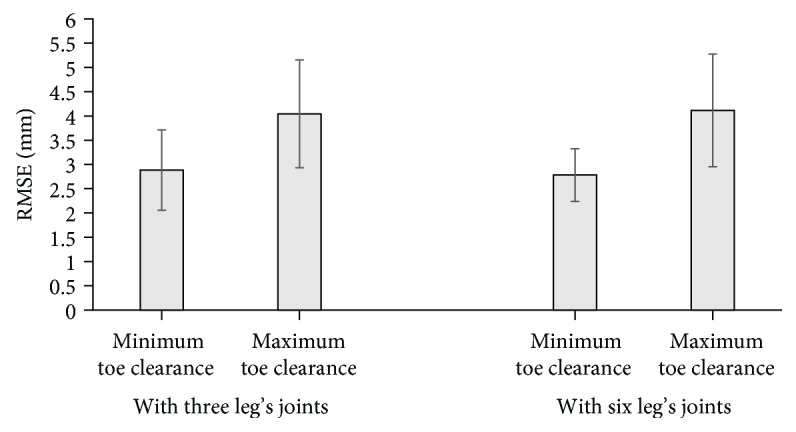
The prediction result using 100 test gait data when the walking velocity changes (RMSE). The values are the mean, and the error var means the standard deviation among the five subjects.

**Figure 14:**
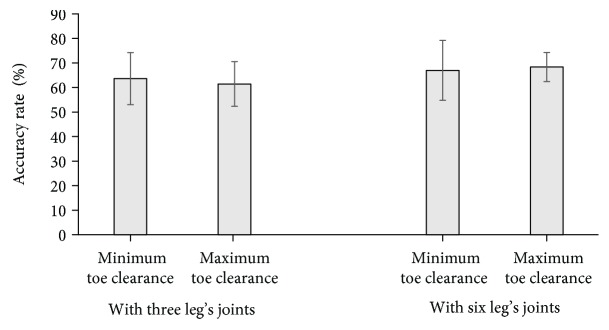
The prediction result using 100 test gait data when the walking velocity changes (accuracy rate). The values are the mean, and the error var means the standard deviation among the five subjects.

Figures 13 and 14 show the RMSE and the accuracy rate of the predicted data for the maximum and minimum toe clearances of 100 test data by training the RBFN using approximately 160 training data in the case of the walking velocity. The prediction error of the minimum toe clearance was lower compared with the previous researches even when the walking speed changed after the RBFN was learned with a constant walking speed. Moreover, the proposed algorithm could detect the value lower than that of the median toe clearance with the probability that was higher than the probability of random detection if walking velocity changed. We assumed that the RBFN parameters reflected the difference of foot kinematics related to the change of the walking velocity because the input data were related to the kinematics of the lower limb. However, the RMSE of the minimum toe clearance and the maximum toe clearance increased when the walking velocity changed. It will be beneficial to train the RMSE with the input data in several conditions for generalized regression. Besides, the standard deviation of the RMSE of all subjects decreased when the left leg joints' information was included as the input values. We assumed that it indicated that more numbers of input parameters related to foot kinematics improved the prediction accuracy. As a future work, we will focus on both the feet and increase of the input parameters of joints of both lower limbs.

The proposed algorithm has an advantage of deriving the toe clearance preliminarily in real time while most previous calculation methods were developed for the estimation of toe clearance [10–14]. Moreover, the prediction accuracy of the proposed algorithm was higher than that of the previous method [15]. Although we normalized the data of the toe clearance for evaluating whether the algorithm could detect the value lower than that of the median toe clearance, the toe height from the ground could be derived because the subtracted value is clear. The proposed system has a limitation because learning is needed in each person, which is similar to previous MTC estimation methods using wearable sensors. Therefore, it requires a learning phase with a camera system before using the algorithm.

The accuracy was lower for subjects whose gait motion and planes in an angular space tended to vary. The angular information always changes with time within one gait cycle. One point on the periodic trajectory in an angular space was extracted in each gait cycle. If phase detection errors occur, it is difficult to compare the articular angle, angular velocity, and angular acceleration differences between the gait cycles. We used the planes of the articular space for the hip, knee, and ankle joints to detect the phase of the angular periodic trajectory. Because the trajectory varied between gait cycles, the planar vectors varied throughout the experiment. The proposed algorithm considered the change of planes by calculating the section plane of the trajectory around the switching points, which was detected by calculating the planes in each gait cycle. However, the phase when the input data were extracted might vary. This study demonstrated that the toe clearance parameters can be predicted using only angular information in the sagittal plane. The accuracy of gait phase detection and the prediction of toe clearance may improve by increasing the input parameters, such as the angles in the coronal plane or the foot contact information.

## 5. Conclusions

This paper proposes a novel toe clearance prediction algorithm with an RBFN using the angles, angular velocities, and angular accelerations of the hip, knee, and ankle joints in the sagittal plane. The proposed algorithm can predict both the maximum toe clearance in the earlier swing phase and the minimum toe clearance in the later swing phase at the same time. The error was 2.99 mm for the maximum toe clearance and 2.34 mm for the minimum toe clearance. Moreover, the root mean square error between the true and predicted values was 4.04 mm for the maximum toe clearance, and 2.88 mm for the minimum toe clearance when the walking velocity changed. The errors of the minimum toe clearance are smaller compared with previous methods. The probability of detecting a value lower than the median toe clearance was higher than 68%; that is, the probability was higher than the probability of random detection. Therefore, a robot using this algorithm may be able to influence the variance of human toe clearance.

In a future work, we will improve the gait phase detection method. Moreover, we will conduct experiments to investigate the effect of robotic assistance with the proposed toe clearance prediction algorithm on older people.

## Data Availability

The data used to support the findings of this study are available from the corresponding author upon request.
